# Investigating the structure of ORTO-15: a meta-analytical simulation study

**DOI:** 10.1007/s40519-018-0621-z

**Published:** 2018-11-29

**Authors:** Radosław Rogoza

**Affiliations:** Cardinal Stefan Wyszyński University, Wóycickiego 1/3, 01-938 Warsaw, Poland

**Keywords:** ORTO-15, Orthorexia nervosa, Measurement, Structure, Monte Carlo simulation

## Abstract

Missbach et al. (Appetite 108:521–524, 10.1016/j.appet.2016.07.010, 2016) argued that there is a critical need to develop new tools assessing orthorexia nervosa (ON), as the existing measure (i.e., ORTO-15; Donini, Eat Weight Disord 10:28–32, 10.1007/BF03327537, 2005) is an unvalidated measure, which fails to adequately assess the prevalence rate of ON. We believe that ignoring past data from ORTO-15 and going in the “baby with the bath water” direction will not catalyse but inhibit ON research. Using data from the review of the psychometric studies analysing the structure of ORTO-15 provided in Missbach et al. (2016), we selected six items, which were present in each study, and estimated effect sizes for the factor loadings. The effect sizes were used in a Monte Carlo simulation study with *N* = 100, 500, and 1000 to test whether the analysed model is valid. The obtained results confirmed that the six-item version of ORTO-15 is a valid and reliable measure of ON. Although new measures of ON are needed, the past data also provide valuable insight into a better understanding of ON.

## Introduction

Orthorexia nervosa (ON) is defined as pathological fixation with healthy eating which shares some common characteristics with eating disorders and obsessive–compulsive disorder [1]. Dunn and Bratman [2] proposed diagnostic criteria defining ON as obsessive focus on “healthy” eating, for which compulsive behaviour and mental preoccupation lead to clinical impairments. Although the construct of ON is well defined [1, 2] and the research in the field is gaining interest (as expressed in the 1180 relevant results in the Google Scholar database for the search “orthorexia nervosa” during the last 5 years), the measurement of ON is open to doubt [3].

The most popular measure of ON, the ORTO-15 [4], which is a 15-item self-report measure on which respondents answer using a 4-point Likert-type scale, was developed as a test for assessment of ON. Using receiver operating characteristic curve analysis, Donini et al. [4] suggested that a threshold of 40 points is optimal in terms of sensitivity and specificity. Whereas the prevalence rate of ON in Donini et al. [4] reached 6.9%, Dunn and Bratman [2] demonstrated, in a review of the literature, that it ranges from 41.9 to 88.7%, which calls into question whether the ORTO-15 is a tool suitable for the assessment of ON. Another weakness of the ORTO-15 [4] is the fact that during its development, the underlying structure of the measure was not assessed and only a three-factor solution was assumed. Five studies investigated the structure of the ORTO-15, and their results were ambiguous, some of them suggesting the presence of one factor [5, 6], some two [7], and some three [8, 9]; moreover, in each study the authors decided to remove some of the items that seemed not to tap the assumed construct, leaving only six items that were in common to all studies.

Missbach et al. [3] argued that the broad range in prevalence rates is rooted in the assessment tool, and they discouraged using the ORTO-15 as a measure to assess ON. While such results clearly provide evidence that diagnosis made on the basis of self-report score from the ORTO-15 is troublesome, we argue that this should not nullify the score’s usage. This is because we believe that ORTO-15 is not a good measure for categorical diagnosis, but it might be good in a dimensional assessment of ON. Such a switch corroborates recent work, for example, that of Clarkin and Livesley [10], which has claimed that all psychological traits, and thus including ON, are in fact dimensional. Missbach et al. [3] also questioned the validity of the ORTO-15, and within the current study we aim to provide evidence from a simulation study that after certain improvements, ORTO-15 may be deemed as a valid measure.

We assume that previous studies were successful in determining which items are suboptimal in the assessment of ON and were systematically excluded. From the initial pool of the 15 items, 6 of them were selected in each study [5–9]; therefore, to highlight the distinctiveness from all previous versions, we label it as ORTO-6. In Table 1, we present the standardized factor loadings of these items originating from *k* = 5 studies with total *N* = 3425, and on the basis of the meta-analysis we provide estimated effect sizes with 95% confidence intervals.

**Table 1:** Standardized factor loadings of ORTO-6 across the studies and the estimated effect sizes

	Model	ORTO3	ORTO4	ORTO7	ORTO10	ORTO11	ORTO12
Missbach et al. [3]	One factor	0.34	0.39	0.37	0.62	0.67	0.66
Varga et al. [6]	One factor	0.82	0.44	0.73	0.38	0.33	0.31
Brytek-Matera et al. [7]	Two factor	0.73	0.38	0.68	0.74	0.53	0.52
Alvarenga et al. [8]	Three factor	0.72	0.71	0.65	0.53	0.57	0.66
Arusoglu et al. [9]	Three factor	0.60	0.61	0.42	0.65	0.52	0.65

Meta-analysis	*d*	95%CI	*Q*	*p*			
ORTO3	0.62	0.60–0.64	297.72	0.001			
ORTO4	0.51	0.49–0.54	96.52	0.001			
ORTO7	0.53	0.51–0.56	169.03	0.001			
ORTO10	0.58	0.56–0.61	84.58	0.001			
ORTO11	0.54	0.52–0.57	94.53	0.001			
ORTO12	0.59	0.56–0.61	122.09	0.001			

*d* the inverse variance weighted mean observed effect size estimate (Hedge’s *g*); *95% CI* lower and upper bounds of the 95% CI for *d; Q χ*^2^ test for the homogeneity of true correlations across studies

## Simulation

On the basis of the estimates from the meta-analysis, nine Monte Carlo simulations were run, each with 1000 replications. The values of items’ residual variances were assumed to be tau equivalent and equalled a quarter. We calculated a separate simulation for the exact, lower-bound, and higher-bound estimates under three conditions regarding the sample size: with 100, 500, and 1000 observations. The data generated from these simulations were the basis for the assessment of the structural validity of the measure. All of the data used for analyses as well as the syntax used for data generation are freely available at https://osf.io/qgs6u.

## Results

Table 2 presents model fit indices and reliability estimates for the measurement model of ORTO-6 under different conditions.

**Table 2:** Model fit indices and reliability estimates of ORTO-6 under different conditions

Estimate	*N*	*χ* ^2^_(9)_	*p*	CFI	RMSEA [90% CI]	*α*
Exact	100	12.96	0.165	0.982	0.066 [0.000–0.140]	0.85
	500	7.75	0.560	10.00	0.000 [0.000–0.045]	0.86
	1000	3.78	0.926	10.00	0.000 [0.000–0.011]	0.87
Lower bound	100	13.03	0.161	0.980	0.067 [0.000–0.141]	0.84
	500	7.76	0.559	10.00	0.000 [0.000–0.045]	0.85
	1000	3.76	0.927	10.00	0.000 [0.000–0.011]	0.85
Greater bound	100	12.87	0.169	0.984	0.066 [0.000–0.140]	0.87
	500	7.48	0.600	10.00	0.000 [0.000–0.045]	0.87
	1000	3.79	0.925	10.00	0.000 [0.000–0.012]	0.88

The results revealed that all of the models, including the lower-bound model, were well fitted to the data and reliable in their measurement. This implies that the ORTO-6 is a structurally valid and reliable measure.

## Conclusion

Missbach et al. [3] stated that previous results of studies obtained from existing ON measures of low quality should be treated with caution. On the basis of conducted simulations, we disagree with such a categorical statement. Indeed, we agree that ORTO-15 (or ORTO-6) is not a valid tool to diagnose ON and to study its prevalence; however, we argue that the tool could be successfully used for the dimensional assessment of ON. Previous research, predominantly using this measure, led to a better understanding of ON, as, for example, such studies provided evidence for its associations with eating disorders and health behaviours, its intensity in dieting styles, or gender differences [1, 4–9]. We agree that there is a need for a tool to assess ON in accordance with the diagnostics criteria [2, 3]; however, we also believe that ORTO-6 might be successfully used as a general marker of orthorexic behaviours used in dimensional diagnosis [10].

## Limitations

The current study provides support for using an abbreviated version of the ORTO questionnaire for the assessment of ON; however, the presented results are neither final nor definitive. Within the simulations, only those items that had not been excluded in several published studies [5–9] were included; however, it is still possible that the analysis carried out on all items would lead to a different item selection. Moreover, the factor loadings included in the meta-analysis were included from different measurement models, which might have influenced the obtained results. Finally, as the assumption of the tau equivalence is seldom met, the structure of the ORTO-6 should be verified by subsequent empirical studies.
